# Molecular Mechanisms of *Clonorchis sinensis*-Host Interactions and Implications for Vaccine Development

**DOI:** 10.3389/fcell.2021.781768

**Published:** 2022-01-18

**Authors:** Stephane Koda, Xing-Quan Zhu, Kui-Yang Zheng, Chao Yan

**Affiliations:** ^1^ Jiangsu Key Laboratory of Immunity and Metabolism, Xuzhou Laboratory of Infection and Immunity, Department of Pathogenic Biology and Immunology, National Experimental Demonstration Center for Basic Medicine Education, Xuzhou Medical University, Xuzhou, China; ^2^ College of Veterinary Medicine, Shanxi Agricultural University, Taigu, China

**Keywords:** *Clonorchis sinensis*, host-worm interactions, immune responses, vaccine, mechanisms

## Abstract

Infections caused by *Clonorchis sinensis* remain a significant public health challenge for both humans and animals, causing pyogenic cholangitis, cholelithiasis, cholecystitis, biliary fibrosis, and even cholangiocarcinoma. However, the strategies used by the parasite and the immunological mechanisms used by the host have not yet been fully understood. With the advances in technologies and the accumulated knowledge of host-parasite interactions, many vaccine candidates against liver flukes have been investigated using different strategies. In this review, we explore and analyze in-depth the immunological mechanisms involved in the pathogenicity of *C. sinensis*. We highlight the different mechanisms by which the parasite interacts with its host to induce immune responses. All together, these data will allow us to have a better understanding of molecular mechansism of host-parasite interactions, which may shed lights on the development of an effective vaccine against *C. sinensis*.

## 1 Introduction

*Clonorchis sinensis* is a liver fluke that is endemic to some parts of Asia: China, South Korea, northern Vietnam, and eastern Russia (Tang et al., 2016). It was estimated that 600 million people were at risk worldwide with an approximate 35 million infected, 15 million of whom were in China (Qian et al., 2016; Harrington et al., 2017). There are several assays for the diagnosis of clonorchiasis including “golden standard” for detection of adult worms or eggs as well as immunological techniques such as enzyme-linked immunosorbent assay (ELISA) (Li et al., 2011; Han et al., 2012). When the presence of *C. sinensis* is confirmed, the treatment consists of the administration of praziquantel (three doses of 25 mg/kg/d praziquantel at 5-hour-interval in 1 day) (Choi et al., 2010). Another option is the use of tribendimidine (400 mg/kg/d per dose), but some side effects have been reported in patients using tribendimidine (Xu et al., 2014). In addition, the intensive and improper usage of these drugs may result in the emergence of resistance to almost all of the anthelmintic drugs (Fairweather et al., 2020). The development of alternative strategies will be urgent since there is no successful vaccine against *C. sinensis* infection. Thus, a good understanding of the mechanisms by which the parasite interacts with its host and the immunological mechanisms used by the host to fight against the parasite may shed light on the development of the strategies to control *C. sinensis* infection. In the present review, we summarize the current status of immunological understanding of *C. sinensis*-host interactions as well as the implications for vaccine development.

## 2 Mechanism of Immunity to *Clonorchis Sinensis*

### 2.1 Host-Worm Interactions-From the Viewpoint of Worms

#### 2.1.1 *C. sinensis* Life Cycle

Liver flukes have a relatively complex life cycle including two intermediate hosts and definitive hosts. For *C. sinensis*, there are two intermediate hosts, namely freshwater snails (*Parafossarulus* sp., *Alocinma* sp., and *Bithynia* sp.) as the first intermediate host and freshwater fish as the second intermediate host, respectively. When the snail takes up the *C. sinensis* eggs, the miracidium grows into sporocyst in the gastrointestinal tract of the snail (Yoshida, 2012). The sporocyst will successively develop into radiae and cercariae, which will be released into the water, swim, and seek the second intermediate host (freshwater fish) (Liang et al., 2009; Yoshida, 2012). In the second intermediate host, the cercariae encyst and develop into metacercaria and take 30–45 days to become matured (Liang et al., 2009). In general, piscivorous mammals including humans get infected with *C. sinensis* by eating raw or undercooked fresh fish containing metacercariae (Lai et al., 2016). Once in the duodenum, the metacercariae excyst and migrate into the bile ducts through the ampulla of Vater within 10–15 min (Kim et al., 2011; Lai et al., 2016). The juveniles then grow into adults in the bile ducts after about 1 month (Yoon et al., 2001).

The components from different developmental stages and locations in *C. sinensis* elicits unique type 1/type 2 immune responses.

**FIGURE 1:**
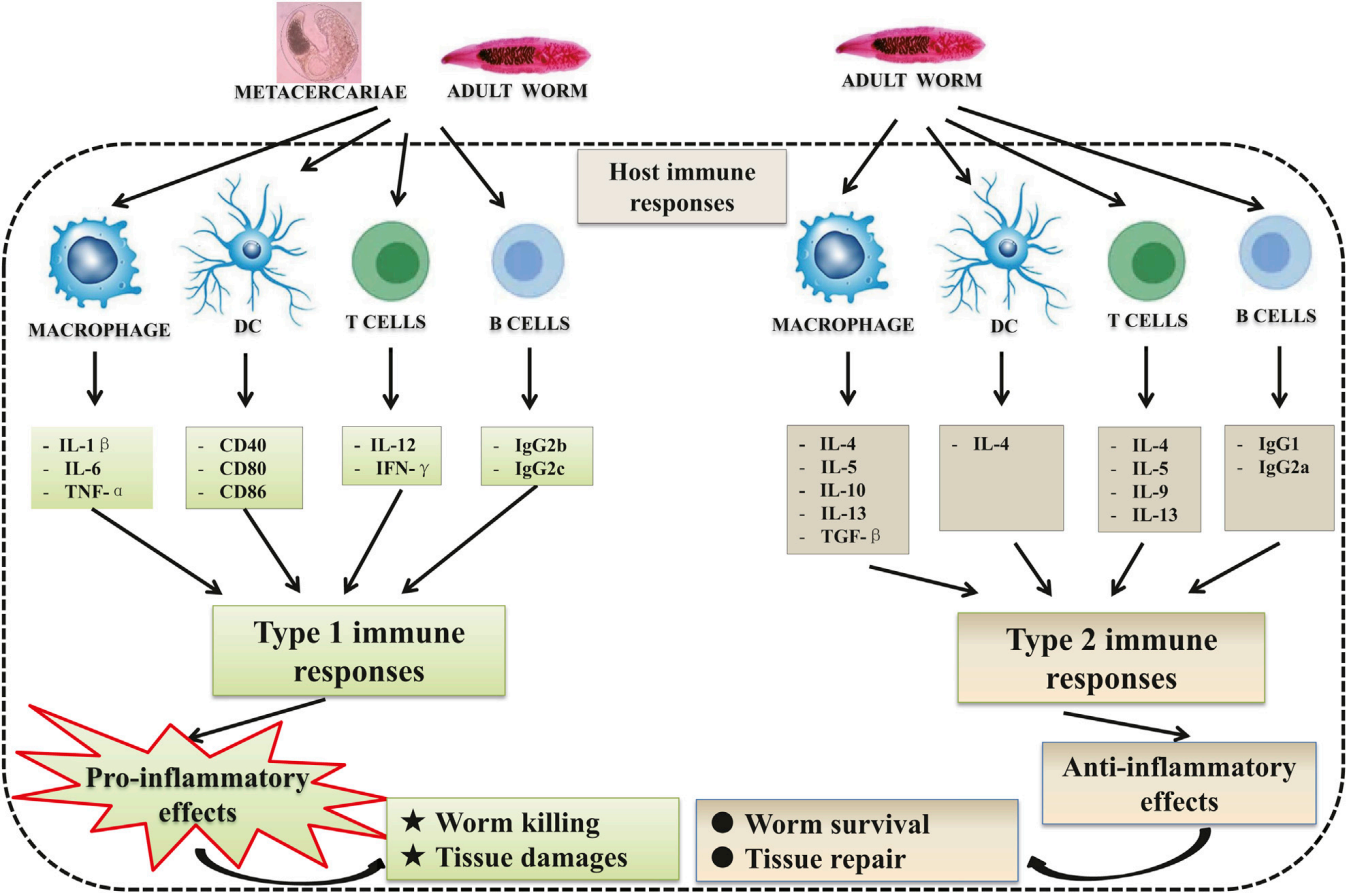
Host immune responses during *Clonorchis sinensis* infection: *C. sinensis* infection leads to the activation of both innate and adaptive immune cells. During its life cycle within definitive hosts, the parasite presents different stages of development leading to a different immune response depending on the stage of development. In the early stage of the infection, the juvenile stages (metacercariae) favor the type 1 immune reaction which is pro-inflammatory with the production of cytokines such as IL-1β, IL-6, and TNF-α by macrophages, and IL-12, IFN-γ and IgG2b, IgG2c by T cells and B cells, respectively. This pro-inflammatory reaction is supposed to lead to the expulsion of the parasite and biliary injuries. Adult parasites, on the other hand, develop strategies that allow them to escape the host immune response. To do this, they trigger the type 2 immune reaction which is anti-inflammatory and pro-fibrotic, with the production of IL-4, IL-5, IL-10, IL-13, and TGF-β by macrophages and T cells, and IgG1, IgG2a by B cells.

*C. sinensis* infections trigger type 1 immune response as well as type 2 immune response (Figure 1). In the early stage of infection, the immune response involved tends to favor the type 1 immune response which is a pro-inflammatory response (Yan et al., 2015a; Kong et al., 2020; Wang et al., 2021). The triggering of type 1 immune response was also observed in patients with acute *C. sinensis* infection (Cai et al., 2004; Cai et al., 2013). The contact of the parasite with its host leads to the production of pro-inflammatory cytokines such as IL-1β, IL-6, TNF-α by macrophages, but also IL-12, and IFN-γ by T lymphocytes (Mao et al., 2015; Kim et al., 2017a; Chung et al., 2017; Bai et al., 2020; Kang et al., 2020). However, with the infection going on, type 2 immune responses (the production of cytokines such as IL-4, IL-13, and transforming growth factor β1) become predominant which can control hyper-inflammation and promote tissue repair (Kim et al., 2017a; Gieseck et al., 2018; Zhao et al., 2018). Furthermore, the worms generally produce compounds that allow them to escape the host’s immune response to avoid the clearance of the parasite by inducing regulatory cytokines such as IL-10 (Xu et al., 2013; Jin et al., 2014; Maizels and McSorley, 2016; Yan et al., 2020a).

**FIGURE 2:**
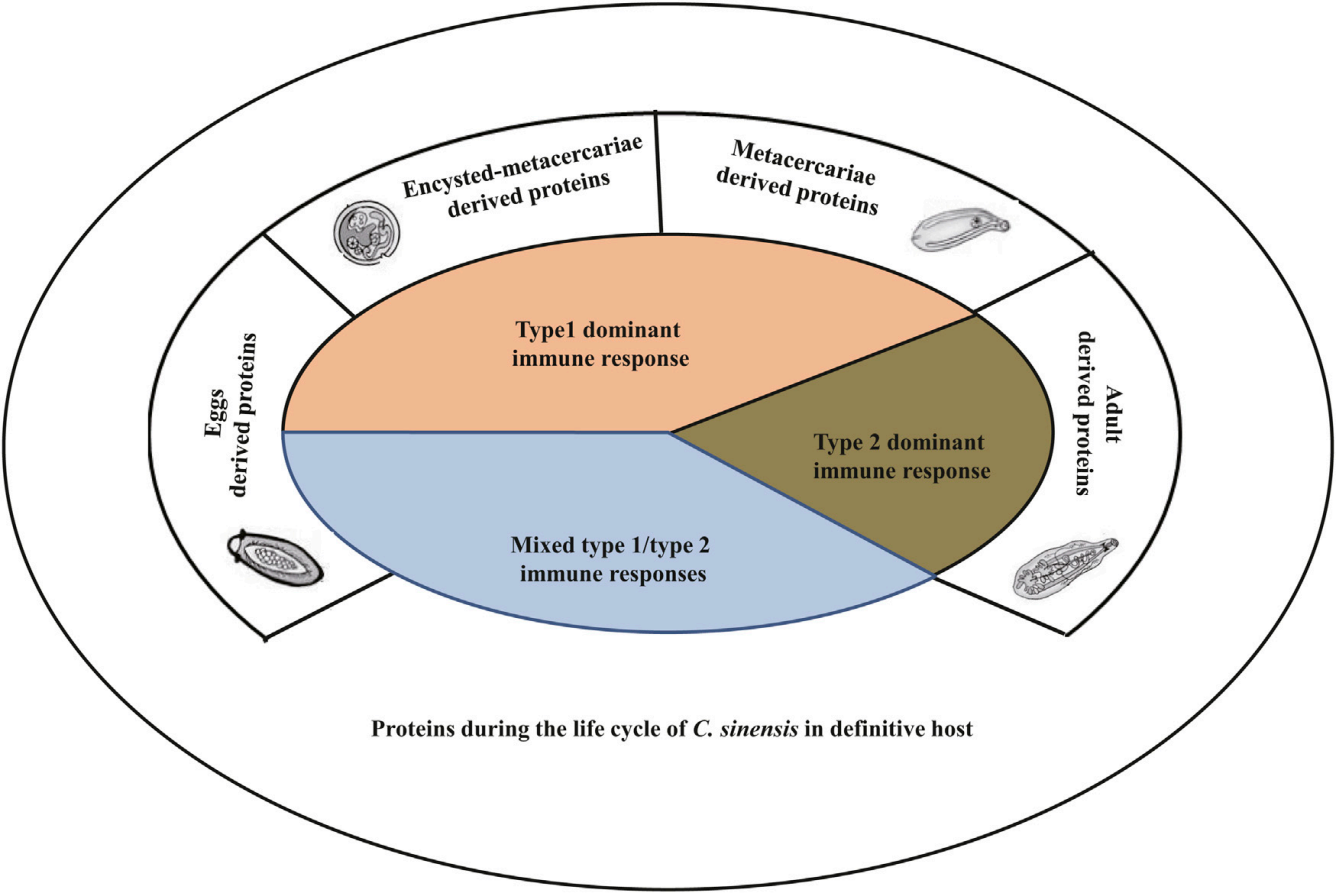
The type of immune responses targeted by *Clonorchis sinensis* components during the life cycle of the parasite within its definitive hosts. During its life cycle in its definitive host, *C. sinensis* produces different types of compounds leading to the activation of either type 1 immune response or type 2 immune response, or both. Typically, proteins excreted by the juvenile stages (encysted-metacercariae and metacercariae) trigger type 1 immune response, while adult parasites secrete compounds that lead to the production of type 2 immune response rather than type 1. Eggs derived proteins generally activate both type 1 and type 2 immune responses.

Actually, the immune responses involved in the host’s defense during infection by *C. sinensis* are quite complicated. This complexity results from the fact that the parasite produces different types of compounds involved in its pathogenicity. Generally, during the life cycle of its definitive host, there are massive compounds of the parasite included excretory-secretory products (ESPs), tegumental proteins (Chung et al., 2018), cyst wall-derived proteins (Wang et al., 2012), egg-derived proteins (Chen et al., 2017), and metabolism-related enzymes among others (Shi et al., 2020). Furthermore, it seems that some compounds excreted by the parasite appear to depend on its stages of development, which implies that the juvenile in the early stages of infection may secrete specific compounds that are different from those excreted in the other stages of infection (Table 1; Figure 2) (Wang et al., 2012; Bian et al., 2014; Mao et al., 2015; Xu et al., 2015; Chen et al., 2017). Although the compounds of *C. sinensis* proteins are complex, the available data showed that recombinant proteins derived from ESPs, tegumental proteins, egg-derived proteins, or proteins extracted directly from ESPs generally and preferentially target a specific immune cell type including innate immune cells such as macrophages, dendritic cells, and/or adaptive immune cells such as T lymphocytes (Table 1) (Habich et al., 2005; Xu et al., 2015; Kim et al., 2017b; Chung et al., 2018; Zhao et al., 2018).

**TABLE 1:** The candidates of potential vaccines against *Clonornchis sinensis*.

Target antigen	Nature of the antigen	Predominant stage of the production	Stimulated targeted cells	Type of the immune response triggered	Cytokines production	Ref.
rCsHSP70	Rec protein	—	Bone marrow dendritic cells	Type 1 immune response	IL-1β, IL-6, and IL-12p70 TNF-α	Chung et al. (2017)
rCsHSP90	Rec protein	—	Bone marrow dendritic cells	Type 1 immune response	IL-1β, IL-6, and IL-12p70 TNF-α	Chung et al. (2017)
CsFHC	Rec protein	Excysted metacercaria, metacercariae and eggs	Hepatic stellate cell	Type 1 immune response	IL-1β and IL-6	Mao et al. (2015)
CsLAP2	Rec protein	Excysted metacercaria	T cells and B cells	Type 1 and Type 17 immune response	IFN-γ, IL-6, IL-10, IL-17A, and TNF-α, IgG1, IgG2a, and IgA	Qu et al. (2014)
CsPK	Rec protein	Eggs	T cells and B cells	Th1-biased immune response	IgG2 and IgG1	Chen et al. (2017)
CsNOSIP	Rec protein	Adult worms (intestine, vitellarium, and eggs)	B cell	Type 2 immune response	IL-4, IL-6, IgG1	Bian et al. (2014)
CsTPs	Rec protein	Adult worms	Bone marrow dendritic cells	Type 2 immune response	IL-4 and IL-13	Zhao et al. (2018)
CsRNASET2	Rec protein	Adult worms	T cells	Th2 immune response	IL-4	Xu et al. (2015)
rCsTegu21.6	Rec protein	Adult worms (tegument)	Dendritic cells and T cells	Type 1/Type 2 immune response	TNF-α, IL-6, IL-1β, IL-10, IL-12p70, IL-2, IL-4, and IFN-γ	Chung et al. (2018)
CsLAP2	Rec protein	Excysted metacercaria (tegument, excretory vesicle)	B cell	Type 1/Type 2 immune response	IgG1 and IgG2a	Deng et al. (2012)
CsPmy	Rec protein	Adult worms, metacercariae	B cell and T cell	Type 1/Type 2 immune response	IgG1 and IgG2a	Wang et al. (2012)
CsTrip	Rec protein	Adult worms (tegument)	CCA cells (HuCCT1)	Type 1/Type 2 immune response	IL-1β, IL-6, TNF-α, IL-10, TGF-β1, and TGF-β2	Pak et al. (2019)
CsLeg	Rec protein	Adult worms	CCA cells (HuCCT1)	Type 1/Type 2 immune response	IL-1β, IL-6, TNF-α, IL-10, TGF-β1, and TGF-β2	Pak et al. (2019)
CsGrb2	Rec protein	Adult worms (oral sucker)	CCA cells (HuCCT1)	Type 1/Type 2 immune response	IL-1β, IL-6, TNF-α, IL-10, TGF-β1 and TGF-β2	Pak et al. (2019)
CsTP 22.3	Rec protein	Adult worms (tegument)	T cells and B cells	Type 1/Type 2 immune response	IgG2a, IgG2c, and IgA	Lee et al. (2017)
CsATP-ε	Rec protein	Excysted metacercaria, adult worms	T cells and B cells	Type 1/Type 2 immune response	IgG1and IgG2a	Lv et al. (2014)

CCA, cholangiocarcinoma; Rec, recombinant; ESPs, Excretory-secretory products.

When we analyze the dualism that exists between type 1 and type 2 immune responses, we can speculate that the parasite controls the immune response to ensure its survival. Seen from this angle, it is important to understand the mechanisms by which the parasite controls this parameter. A thorough analysis of the currently available data on the compounds excreted/extracted from the parasite has allowed us to observe that the abundances of components of the parasite are various due to different developmental stages of *C. sinensis* (Table 1). Some compounds are excreted during the whole life cycle of the parasite, however, the quantities vary from one developmental stage to another; other compounds, on the other hand, are excreted during certain stages of the life cycle, and either at the metacercaria stage or at the adult worm stage (Table 1; Figure 2). Indeed, compounds excreted during a certain developmental stage of the life cycle generally trigger a specific type of immune response; either type 1 for proteins excreted by the larval and juvenile stages and type 2 for adult parasites or a mixed type 1/type 2 for proteins produced during the entire life cycle (Table 1). For example, Mao et al. studied the heavy chain ferritin protein extracted from *C. sinensis* (CsFHC) and demonstrated that this protein is excreted throughout the parasite’s entire life cycle (Mao et al., 2015). However, the results revealed that during the egg, encyst metacercarial and metacercarial stages, the amount of protein excreted was significantly higher than that during the adult worm stages (Mao et al., 2015). Furthermore, this protein triggered the type 1 immune response rather than type 2 with an increase in the production of pro-inflammatory cytokines such as IL-1β and IL-6 in a NF-κB-dependent manner (Mao et al., 2015). Similarly, Chen et al. (2017) revealed that *C. sinensis* pyruvate kinase (CsPK), which was expressed in large quantities in the egg, encyst and metacercarial stages, triggered a type 1 immune response (Chen et al., 2017). Concerning the proteins extracted/secreted from adult worms, almost all of these proteins seem to trigger the type 2 immune response (Table 1). Some proteins are excreted during the several stages of life cycle without any significant difference in the amount of proteins excreted from one stage to the other stages. This is the case of the leucine aminopeptidase 2 (LAP2) protein gene of *C. sinensis* which triggered the mixed type 1/type 2 immune response (Deng et al., 2012). Someother proteins produced during the several stages of life cycle, such as *C. sinensis* paramyosin (CsPmy) (Wang et al., 2012), ATP synthase subunit ε-like protein of *C. sinensis* (CsATP-ε) (Lv et al., 2014) excreted during the entire cycle triggered a mixed Th1/Th2 immune response.

In addition, the amount of proteins excreted depends on the localization of its production. Thus, certain proteins are produced by the parasite’s teguments and are rapidly excreted, and allowing the parasite to interact rapidly with its host. Several pieces of evidence have demonstrated that proteins produced by external organs such as the tegumental proteins, the cell wall proteins trigger the mixed type 1/type 2 immune response. Work carried out on tegumental protein derived from *C. sinensis* (rCsTegu21.6) (Chung et al., 2018), *C. sinensis* tegumental protein 22.3 kDa (CsTP 22.3) (Lee et al., 2017), *C. sinensis* paramyosin (CsPmy) (Wang et al., 2012) from the cyst wall have all shown the involvement of these proteins in triggering the type 1/type 2 immune response in the host. Taking all these parameters into consideration, we speculate that proteins and compounds excreted rapidly by the larval stages and juvenile forms of the parasite possibly contribute to the triggering of type 1 immune reaction, while compounds excreted by adult parasites preferentially trigger type 2 immune reaction (Figure 2; Table 1).

### 2.2 Host-Worm Interactions-From the Viewpoint of the Host

#### 2.2.1 The Involvement of Immune Cells in the Pathogenesis of *C. sinensis*

The understanding of the immune responses induced by *C. sinensis* is important for the development of vaccines against the parasite. Different effectors cells are involved in the pathogenicity of *C. sinensis*.

##### 2.2.1.1 Macrophages

Macrophages are the key component in the initiation of the immune response, as antigen-presenting cells, macrophages play a very important role in triggering the activation of adaptive immune cells (Murray and Wynn, 2011; Shan and Ju, 2020). Several studies have demonstrated the role of macrophages in *C. sinensis* infection. Treatment of macrophages with *C. sinensis* ESPs has been shown to induce the differentiation of macrophages toward classically activated macrophages (M1) *in vitro* and the production of pro-inflammatory cytokines (Kim et al., 2017a; Kang et al., 2020). These authors also showed that infection of mice resulted in mixed M1 and alternatively activated macrophages (M2) (Kim et al., 2017a). The percentage of M1 and M2 macrophages were different according to the stage of the infection with the increase in M1 macrophages in the early stage of infection, while the M2 macrophages were predominant in the late stage of the infection especially in the fibrotic part (Kim et al., 2017a; Koda et al., 2021). Besides, the various components of *C. sinensis* induces the differentiation of macrophages to both M1 and M2 macrophages (Wi et al., 2012; Kim et al., 2017a; Yan et al., 2020a; Kang et al., 2020; Koda et al., 2021). Moreover, it has been shown that macrophages are associated with the resistance of mice to the second infection by *C. sinesis*, which showed the decrease of IL-10 and IL-13 produced by M2 macrophages and increases of specific IgE, IgG1, and IgG2a levels in serum at 1 week or 4 weeks following re-infection (Kim et al., 2017b). The autonomic nervous system has been studied for its involvement in the control of macrophages activation in recent years (Lorton and Bellinger, 2015; Stakenborg et al., 2020). The role of beta 2 adrenergic receptors (β2-AR, a kind of guanine nucleotide-binding G protein–coupled receptor that can bind with norepinephrine and epinephrine to induce diverse physiological effects) have also been shown to regulate the activation of M2 macrophages during *C. sinensis* infection in our recent study (Koda et al., 2021). It was showed that deletion of *Adrb2* (encoding β2-AR) was associated with a decrease in liver fibrosis and the decrease in the infiltration of M2 macrophages in *C. sinensis* infected mice (Koda et al., 2021). Moreover, the *in vitro* study showed that β2-AR promoted M2 activation mediated by mTORC1 since inhibition of mTORC1 by rapamycin significantly decreased M2 markers in macrophages isolated from β2-AR defective mice (Koda et al., 2021).

##### 2.2.1.2 Dendritic cells

DCs are professional antigen-presenting cells and play a very important role in the activation of the adaptive immune response (Motran et al., 2018). Bone Marrow-Derived Dendritic cells (BMDCs) stimulated with *C. sinensis* ESPs decreased the high levels of IL-12 but increased the levels of IL-10 that was induced by LPS, which suggested that *C. sinensis* ESPs may induce an anti-inflammatory responses in DCs (Hua et al., 2018). The immunization of mice with the recombinant HSP70 and HSP90 from *C. sinensis* (rCsHSP70 and rCsHSP90) also induced a predominant type 1 immune response (such as IL-1β, IL-6, IL-12p70, and TNF-α) (Chung et al., 2017). However, stimulating DC2.4- a mouse cell DC line with crude antigens of *C. sinensis*, Jin et al. found that DCs were activated to produce high levels of IL-10 and TGF-β via activation of ERK1/2 (Jin et al., 2014).

##### 2.2.1.3 Lymphocytes

Early studies have investigated the rat lymphocyte proliferation, differentiation, and cytokine production in response to *C. sinensis* infection (Quan et al., 2002). The *in vitro* stimulation of splenic lymphocytes (SLC) and mesenteric lymph node cells (MLNC) of rats infected with *C. sinensis* with mitogen phytohaemagglutinin (PHA), *C. sinensis* ESPs, *C. sinensis* crude antigen, and *Anisakis* larvae antigen revealed that the *C. sinensis* ESPs and crude antigen could potently induce the proliferation of lymphocyte and the lymphocyte proliferation in MLNC was higher than that in SLC (Quan et al., 2002). These results suggest that the vaccine candidate that may induce the increase in the gastro-intestinal immune response could provide a strong immune response against *C. sinensis*. *C. sinensis* infection also induced the activations of CD4^+^ lymphocytes including Th1/Th2/Th17 and Treg in FVB mice (Kong et al., 2020); interestingly, it was found that the increases of Th2 and Treg subpopulations were positively correlated with severe biliary fibrosis in FVB mice caused by *C. sinensis*, compared with BABL/c mice which had less biliary injuries and fibrosis (Zhang et al., 2017). A recent study revealed that the immunization of mice with a CsAg17 protein and CsAg17 cDNA resulted in a significant reduction of the worm burden (64 and 69%, respectively); the immunized mice showed a significant increase in the recruitment of CD4^+^ and CD8^+^ T cells as revealed by the increase of the proportion of CD3^+^/CD4^+^ and CD3^+^/CD8^+^ T cells (Bai et al., 2020). Moreover, the production of type 1 cytokines such as IL-2, IL-12, and IFN-γ was also increased in immunized mice 2 weeks after immunization, resulting in reduced liver damage compared to non-immunized mice in that study (Bai et al., 2020).

Natural killer T cells (NKT) cells are the subpopulation of T cells that express both markers of NK cells and lymphocytes (Brennan et al., 2013). Some pieces of evidence have suggested that NKT cells may be involved in the pathogenesis of *C. sinensis* which was related to the susceptibility of the different strains of mice: it showed that compared to FVB mice, BALB/c mice infected with *C. sinensis* displayed fewer liver damages as well as liver fibrosis and necrosis of hepatocytes, which was accompanied with less population of hepatic DX5^+^ NKT cells (Zhang et al., 2017; Bai et al., 2021). On the other hand, NKT is mostly associated with the production of a pro-fibrotic cytokine such as IL-4 and IL-13, which may play an important role in liver fibrosis caused by *C. sinensis* infection (Gao and Radaeva, 2013; Mathews et al., 2016).

### 2.3 The Imbalance of Treg/Th17 Contribute to the Pathogenesis of *C. sinensis*

It has been previously demonstrated that Treg cells are kinds of regulatory cells and play a role in maintaining self-tolerance, immunologic homeostasis, and supressing inflammatory responses in infectious diseases and autoimmune diseases (Sakaguchi, 2004; Sakaguchi, 2005). However, Th17 is associated with the immunopathology of infectious diseases and plays an important role in the fighting against extracellular pathogens (Chen et al., 2013). Indeed, inflammatory and auto-immune diseases which are characterized by sustained inflammation can induce tissue damage, thus the balance of Treg/Th17 is crucial to control the excessive inflammatory reaction (Naufel et al., 2017; Xia et al., 2018). In helminth infection, Treg cells can favor the parasite survival and increase the pathological damages induced by the parasites. In the early stage of infection of BALB/c mice with *C. sinensis* (14 days post-infection), the inflammation was increased to promote the expulsion of the parasite, while the level of Treg/Th17 remained low (Yan et al., 2015c). At days 28 and 56 post-infection, the level of Treg/Th17 was significantly increased in infected mice compared to that of control mice, while the level of inflammatory cells was decreased, thus the increase in Treg/Th17 was associated with the degree of pathological damages, suggesting that the imbalance of Treg/Th17 in *C. sinensis* promotes the progression of liver damages induced by the parasite (Yan et al., 2015c).

### 2.4 The Molecules and Signaling Pathways Involved in Immune Responses During *C. sinensis* Infection

#### 2.4.1 The Role of Toll-like Receptors

**FIGURE 3:**
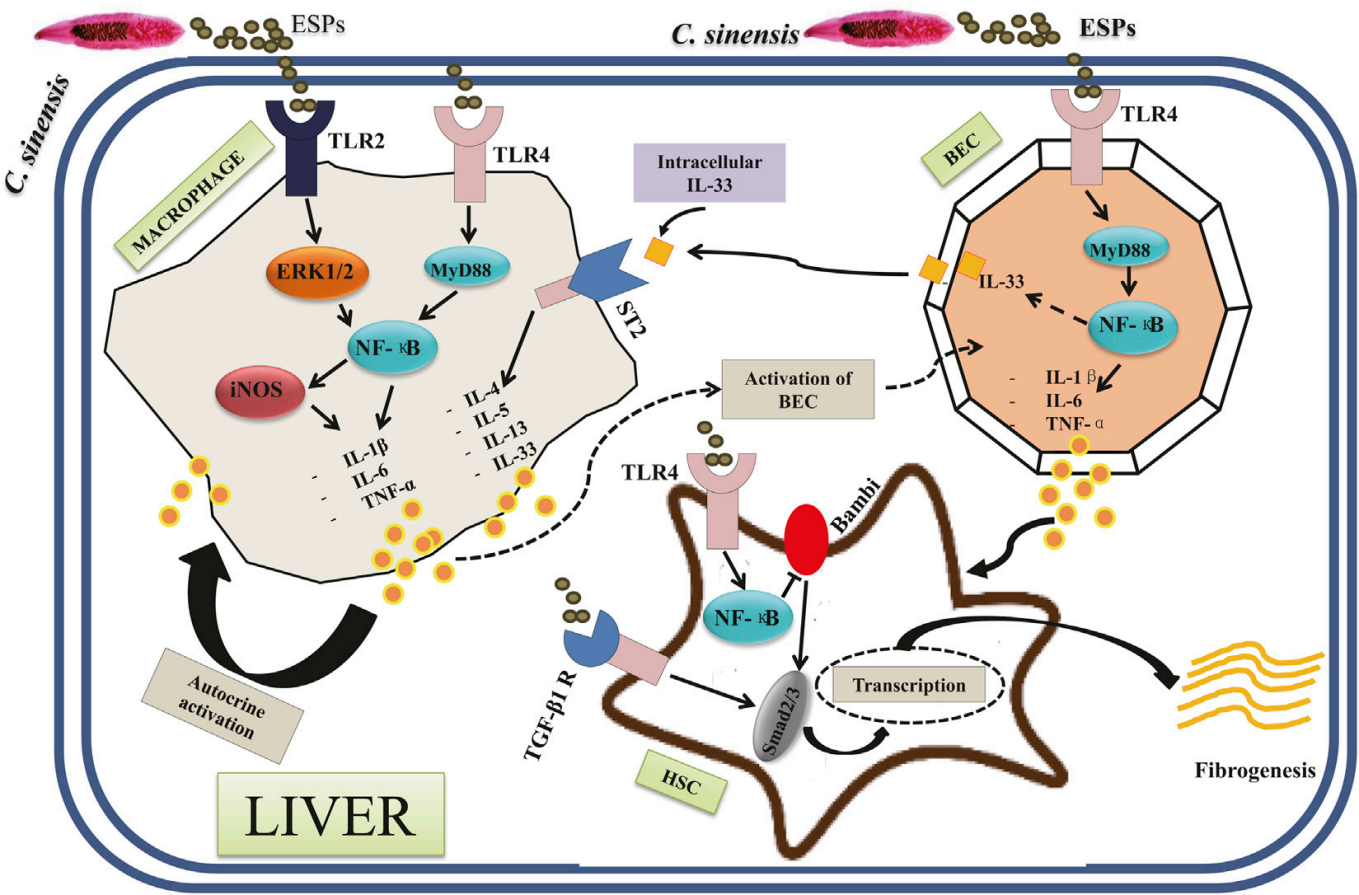
Different types of signaling pathways involved in the pathogenesis of *Clonorchis sinensis* infection: The interaction between the parasite and its host results in the activation of several signaling pathways. The activation of TLR2 and TLR4 in macrophages by *C. sinensis* ESPs leads via ERK1/2 and MyD88, respectively, to the activation of NF-κB. The transcription leads to the production of pro-inflammatory cytokines (IL-1β, IL-6, and TNF-α). The activation of the TLR4 of cholangiocytes also leads to the production of the pro-inflammatory cytokines (IL-1β, IL-6, and TNF-α) via the MyD88 and NF-κB signaling pathways. Another signaling pathway involves the TGF-β1 receptor in hepatic stellate cells. In this signaling pathway, the activation of TGF-β1 R by *C. sinensis* ESPs leads to the activation of Smad2/3 followed by transcription with an increase in cell growth and survival. In hepatic stellate cells, the activation of TLR4 also leads to the activation of NF-κB and Smad2/3 with an increase in cell growth and survival.

Figure 3 shows the current knowledge of the molecules and signaling pathways of immune responses involved in the interactions between *C. sinensis* and its host. Basically, in the liver cholangiocytes are the first line of defense against microorganisms from the duodenum (Banales et al., 2019). Because of this characteristic, cholangiocytes behave like innate immune cells (Harada and Nakanuma, 2012). Cholangiocytes possess several Pattern Recognition Receptors (PRRs) such as Tolls Like Receptors (TLRs) that allow them to sense when they are in contact with Pathogen-Associated Molecular Patterns (PAMPs) such as *C. sinensis* itself and the compounds excreted by the parasite (Nakanuma et al., 2001; Yan et al., 2015b; Yan et al., 2020a). A previous study has demonstrated that the activation of TLRs located in cholangiocytes mainly leads to the production of pro-inflammatory cytokines such as IL-1β, IL-6, IL-8, and TNF-α, thus representing type 1 dominant immune response (Yan et al., 2020b). It is, therefore, reasonable to hypothesize that activation of TLRs by compounds from *C. sinensis* may lead to type 1 dominant immune response. Indeed, TLR4 plays an important role in *C. sinensis* infection, notably in the production of pro-inflammatory cytokines, and the maturation of dendritic cells (Hua et al., 2018). A recent work has shown that during *C. sinensis* infection, infected mice showed significant activation of TLR4 with the production of pro-inflammatory cytokines IFN-γ, IL-6, and TNF-α, but also Treg cytokines such as IL-10 (Hua et al., 2018). Furthermore, these data revealed that treatment of BMDCs with ESPs of *C. sinensis* associated with TLR4 ligand LPS resulted in accelerated maturation of CDs (CD80, CD86, and MHC II). Yan et al. highlighted the important role played by TLR4 on the production of pro-inflammatory cytokines during *C. sinensis* infection. They showed that treatment of bile epithelial cells with *C. sinensis* ESPs resulted in significant activation of TLR4 as well as the proteins of its downstream signaling pathways including the adaptive protein MyD88 and transcription factor NF-κB (p65). Furthermore, the production of TNF-α was increased significantly when BECs were stimulated with ESPs, while the inhibition of TLR4 by a peptide -VIPER resulted in a reduction of TNF-α production (Yan et al., 2015b).

TLR2 also plays an important role in *C. sinensis* infection. Although only limited work has been done on the role of TLR2, Shen et al. have shown that activation of TLR2 correlates with increased susceptibility to liver damage induced by *C. sinensis* (Shen et al., 2017). Indeed, TLR2 plays a role in the regulation of iNOS/NO and significant production of iNOS/NO could influence the production of the anti-inflammatory cytokines IL-10 and TGF-β by a mechanism that remains unknown (Yang et al., 2017). A recent work conducted by our research group showed that the recombinant protein from *C. sinensis* (rCsHscB) activated TLR2/ERK1/2 signaling pathway to lead to a high production of IL-10 (Yan et al., 2020a). rCsHscB may act as a TLR2 agonist to activate macrophages and the production of IL-10 (Yan et al., 2020a).

### 2.5 IL-33/ST2 Signaling Pathway

Interleukin-33 (IL-33) belong to the member of the IL-1 family cytokine. IL-33 plays a role in the regulation of the Th2 immune response via the ST2 chain transmembrane, notably by driving the production of type 2 cytokines such as IL-4, IL-5, and IL-13 (Liew et al., 2010). Some studies have also shown that IL-33 can induce the production of IFN-γ in animal models of experimental autoimmune encephalomyelitis (EAE) (Li et al., 2012), suggesting that IL-33 can induce type 1 as well as type 2 depending on the type of stimuli. Concerning *C. sinensis* infection, Yu et al. (Yu et al., 2016) revealed that IL-33/ST2 induces a strong activation of type 2 immune response on CD4^+^ T cells, but not on CD8^+^ T cells with the production of type 2 cytokines such as IL-4, IL-5, and IL-13. However, the involvement of IL-33/ST2 signaling on the activation of type 1 immune response in *C. sinensis* infection requires further investigation.

### 2.6 Involment of Mannose Receptor

Mannose receptors (MR) are receptors belonging to the members of the type I C-type lectin receptor and interacts with glycans which requires calcium (Figdor et al., 2002). MR has been aptly studied on dendritic cells (DCs). As antigen-presenting cells, DCs are able to recognize antigens of different sources from helminths (Maizels and McSorley, 2016; Motran et al., 2018). Indeed, certain compounds promote the maturation of DCs leading to the production of type 1 cytokines such as IL-12 and IFN-γ (Hua et al., 2018). Other compounds, on the other hand, prevent the maturation of DCs that promote the production of type 2 cytokines (Hua et al., 2018). Zhao et al. showed that the *C. sinensis* adult-derived proteins (CsTPs) supressed the maturation of bone marrow-derived Dendritic Cells (BMDC) caused by LPS via MR *in vitro* and promoted differentiation of naive T cells into Th2 cells presented by BMDC and secretion of the type 2 cytokines such as IL-13 and IL-4 (Zhao et al., 2018). In addation, they showed that the CsTPs protein exerts its effect on DCs via MR but not through TLR2, TLR4, DC-SIGN, and Dectin-2, thus highlighting the role played by MR on immune cells in the presence of the *C. sinensis* component (Zhao et al., 2018).

## 3 The State of Art in the Development of a Vaccine Against *C. Sinensis*

The use of vaccines to fight *C. sinensis* may considerably reduce the impact of this parasite on the public health, which requires a better understanding of interaction between *C. sinensis* and its host. However, no successful vaccine is currently available for fighting *C. sinensis* infection, but some efforts have been made to investigate the promising vaccines to fight against *C. sinensis*. There has been significant progress in the identification of effective vaccine candidates to control liver flukes (summarized in Table 1). However, for the time being, most of these works are at experimental stages but are nevertheless an important element for future marketable vaccines. In the following, we summarize the progress in developing vaccines against *C. sinensis*, which may provide the prospective for future successful development of vaccines against liver flukes.

### 3.1 Recombinant Proteins

Recombinant protein vaccines are a technology that relies on the ability of one or more combined antigens to induce an immune response. This form of vaccine has the advantage of avoiding certain problems with macromolecular vaccines such as the risk of co-purification and undesirable contaminants (Cox, 2012; Perera and Ndao, 2021). Another fundamental problem overcome by this technology is the complexity of obtaining sufficient quantities of purified antigenic components (Lauer et al., 2017). Several recombinant proteins have been attempted as potential vaccine candidates against liver flukes.

As with all helminths, during *C. sinensis* infection, the adaptive immune response plays a major role in the defense process. The parasite causes the activation of the cellular as well as the humoral immune response. In the work carried out by Wang et al. (2012), the immunized mice showed an estimated 54.3% reduction of worm burden with the activation of type 1/type 2 immune response and production of IgG1/IgG2a subtypes in serum. Using the same protein (paramyosin from *C. sinensis*), Sun et al. (2018) demonstrated that immunization of mice with recombinant paramyosin (B.s-CotC-CsPmy) induced significant production of IgG1 and IgG2a increased from week 2 to week 6 post-immunization, indicating that combined type 1/type 2 immune responses were successfully provoked by B.s-CotC-CsPmy recombinant spores (Sun et al., 2018). Many other works such as those of Zhou et al.*,* 2007; Wang et al., 2014 showed relatively moderate protection estimated at 56.29 and 44.7% respectively, implying mainly production of mixed IgG1/IgG2a immune response. Vaccine trials on helminths infection have sufficiently demonstrated that activation of B and T lymphocytes promotes a better immune response. For example, the immunization of rats with baculovirus expressing CsTP 22.3 from the tegument of *C. sinensis*, and virus-like particles (VLP) containing CsTP22.3 evoked systemic IgG (IgG1 and IgG2c) in sera and mucosal IgA antibodies (feces and intestines) as well as T cell immune responses, resulting in stronger protection estimated at more than 70% (Lee et al., 2017).

Although the adaptive immune response plays a major role in defense during *C. sinensis* infection, the innate immune response plays an important role in triggering the immune response (de Veer et al., 2007; Flynn et al., 2010). The use of recombinant proteins for vaccine development should take into account the ability of the target protein to activate both innate and adaptive immunity. A study has shown that the recombinant 21.6 kDa tegumental protein derived from *C. sinensis* was able to activate both innate and adaptive immune cells (Chung et al., 2018). Mice immunized with rCsTegu21.6 showed an acceleration of expression of co-stimulatory molecules (CD40, CD80, and CD86) on dendritic cells, and an increase in pro-and anti-inflammatory cytokine production (Chung et al., 2018). Besides, the stimulation of T cells with rCsTegu21.6 resulted in the production of cytokines such as IL-2, IL-4, and interferon (IFN)-γ *in vitro*, which are essential in inducing humoral immunity for defense against parasitic helminths (Chung et al., 2018).

### 3.2 DNA Vaccines

For many years, conventional vaccine approaches have consisted either of using specific antigen that can cause a reaction of the host’s immune system against a given pathogen or of inoculating attenuated or weakened pathogens that also have immune-stimulating properties of the host’s immune system (Gause et al., 2017). DNA vaccination has evolved as an alternative approach to vaccine development (Li and Petrovsky, 2016). Although the immunological mechanisms involved during DNA vaccination were poorly known in *C. sinensis* infection, DNA vaccine has several advantages compared with the traditional methods including the stimulation of both B- and T-cell responses, increased vaccine stability without any infectious agent, and the relative ease of large-scale manufacture (Jayaraj et al., 2019). However, DNA vaccination has the disadvantage of being less immunogenic than recombinant protein-based vaccines (Jayaraj et al., 2012).

DNA-based vaccines are generally used in viral infections because of their ability to activate T-cells and trigger the type 1 dominant immune response (Lee et al., 2006). However, during helminth infection, the immune mechanism requires the activation of type 1 as well as type 2 immune response and the intervention of cellular and humoral immune response (Diemert et al., 2018). Lee et al. (2006) developed a DNA-based vaccine encoding *C. sinensis* cysteine proteinase (pcDNA3.1-CsCP), which was only able to induce a low level of protection estimated at 31.5%, lower than the protection induced by almost all the recombinant proteins, possibly because vaccination with this DNA vaccine activated only type 1 immune response rather than type 2 (Lee et al., 2006). In another work, Wang et al. also highlighted the weak protection induced by DNA vaccines (Wang et al., 2012). In their comparative study of the efficacy of the recombinant protein and nucleic acid of *C. sinensis* paramyosin (CsPmy) as a potential vaccine candidate, they found that both (recombinant protein and nucleic acid) showed strong immunogenicity and triggered mix type 1/type 2 immune responses, as evidenced by persistently increased antibody titers and increased level of IgG1/IgG2a subtypes in serum. Aslo, the recombinant protein resulted in an estimated protection of 54.3%, significantly higher than that provided by the DNA-based vaccine which was only 36.1% (Wang et al., 2012).

### 3.3 Vaccines that Protect the Intermediate Hosts

An alternative strategy to develop successful vaccines against *C. sinensis* infection is to immunize intermediate hosts since it can intercept the spread of the liver flukes to humans and animals effectively. In a recent study, Jiang *et al.* immunized grass carps (*Ctenopharyngodon idella*), a main second intermediate host for *C. sinensis,* and with a recombinant *C. sinensis* protein (PEB03-CsENO) delivered by spores of *B. subtilis* (Jiang et al., 2017)*.* This immunization induced both systemic and local mucosal immune responses, aroused reactive type 1/type 2 immune response, and also enhanced the non-specific immune response (Jiang et al., 2017). In addition to CsENO, recombinant paramyosin and cysteine protease of *C. sinensis* delivered by spores of *B. subtilis* were also evaluated in grass carps, showing that these vaccine candidates induced sufficient mucosal and humoral responses to protect against metacercaria of *C. sinensis* in these fish (Tang et al., 2017; Sun et al., 2020).

## 4 Concluding Remarks and Future Perspectives

As discussed above, although some progress in the studies of host-parasite interactions and mechanisms of immune protection have been uncovered, it still lacks enough knowledge of pathogenesis caused by this liver fluke, and knowledge of immune responses is needed for protection from worm infection. Firstly, unlike viruses, bacteria, fungi, and even protozoa, many antigens expressed by worms are “immune tolerant”, which can induce only low or no response to a vaccine in a host, but the understanding of the mechanism underlying are largely unknown. Second, the hepatobiliary system has unique immunological properties as it has particular subsets of immune cells and immune molecules due to its special structures and microenvironment (Varol et al., 2015; Doherty, 2016). There are gaps of precise understanding of host-parasite interaction in the liver, which should be taken into consideration during the development of vaccines against *C. sinensis*. To the best of our knowledge, there are no vaccine candidates against *C. sinensis* that are currently advancing to the stage of a clinical trial, thus the discoveries of new promising vaccine candidates are being investigated in the laboratories. Fortunately, the application of new technologies such as “omics” technology, gene-editing technology, and machine-learning-based reverse vaccinology undoubtedly will accelerate the progress of the further investigation of worm-host interactions as well as identification of vaccine candidates against *C. sinensis,* although some hurdles should be overcome. Given these perspectives, the prospect of successful development of vaccines against *C. sinensis* infection appears bright, although there is still a long way to go.
